# Avian Bornaviruses in Wild Aquatic Birds of the Anseriformes Order in Poland

**DOI:** 10.3390/pathogens11010098

**Published:** 2022-01-15

**Authors:** Edyta Świętoń, Kamila Dziadek, Krzysztof Śmietanka

**Affiliations:** Department of Poultry Diseases, National Veterinary Research Institute, Al. Partyzantów 57, 24-100 Pulawy, Poland; kamila.dziadek@piwet.pulawy.pl (K.D.); ksmiet@piwet.pulawy.pl (K.Ś.)

**Keywords:** aquatic bird bornavirus, wild birds, phylogenetic analysis

## Abstract

Bornaviruses are a diverse family of viruses infecting various hosts, including birds. Aquatic bird bornavirus 1 (ABBV-1) and aquatic bird bornavirus 2 (ABBV-2) have been found in wild waterfowl but data on their prevalence are scarce. To gain knowledge on the occurrence of ABBVs in Poland, samples originating from dead birds of the Anseriformes order collected in 2016–2021 were tested with a real time RT-PCR method targeting the ABBVs genome. A total of 514 birds were examined, including 401 swans, 96 ducks and 17 geese. The presence of ABBV-1 RNA was detected in 52 swans (10.1% of all tested birds) from 40 different locations. No positive results were obtained for ducks and geese. Sequences of about 2300 bases were generated for 18 viruses and phylogenetic analysis was performed. A relatively low genetic diversity of the examined ABBV-1 strains was observed as all were gathered in a single cluster in the phylogenetic tree and the minimum nucleotide identity was 99.14%. The Polish strains were closely related to ABBV-1 identified previously in Denmark and Germany, but a limited number of sequences from Europe hinders the drawing of conclusions about interconnections between Polish and other European ABBVs. The results of the present study provide new insights into the distribution and genetic characteristics of ABBVs in wild birds in Europe.

## 1. Introduction

Bornaviruses, members of the *Bornaviridae* family, are found in multiple host species, including birds, mammals and reptiles. Their genome, in a form of a single-stranded negative-sense RNA of about 9000 nucleotides, encodes six proteins: nucleoprotein (N), small accessory protein (X), phosphoprotein (P), matrix protein (M), surface glycoprotein (G) and large protein (L) [1].

Bornaviruses infecting birds belong to five out of eight species within the *Orthobornavirus* genus (*Psittaciform 1 orthobornavirus*, *Psittaciform 2 orthobornavirus*, *Passeriform 1 orthobornavirus*, *Passeriform 2 orthobornavirus*, and *Waterbird 1 orthobornavirus*) [1]. Species *Psittaciform 1 orthobornavirus* comprises six parrot bornaviruses (PaBV-1, -2, -3, -4, -7 and -8), and PaBV-5 and -6 belong to species *Psittaciform 2 orthobornavirus*. Bornaviruses found in passerine birds include canary bornavirus 1 to 3 (CnBV-1, -2, -3), and munia bornavirus 1 (MuBV-1) (species *Passeriform 1 orthobornavirus*) and estrildid finch bornavirus 1 (EsBV-1) (species *Passeriform 2 orthobornavirus*). Species *Waterbird 1 orthobornavirus* comprises aquatic bird bornavirus 1 and 2 (ABBV-1 and -2) [1].

The clinical disorders associated with bornavirus infection have been well documented in psittacine birds, in which proventricular dilatation disease (PDD) is a common outcome [2]. The virus causes damages in neurons of the enteric and central nervous system, resulting in an impairment of the gastrointestinal tract function accompanied often by neurological signs [3]. A similar disease was also reported in captive passerine birds, e.g., canaries [4].

Aquatic birds of the Anseriformes order have been shown to be the main hosts for the members of the species *Waterbird 1 orthobornavirus* (ABBV-1 and -2) [1]. ABBV-1 was first identified in North America [5] and subsequently was shown to be widespread in wild geese, swans and gulls [6,7,8,9,10]. ABBV-2 has been detected so far only in wild ducks in the USA [11]. Data on the prevalence of bornaviruses in European wild birds are more limited as there are only reports on the detection of ABBV-1 in Denmark and Germany, mainly in wild geese and mute swans [12,13]. Whether the infection of aquatic birds with ABBVs has any clinical implications remains elusive. Based on clinical signs, histopathology and immunohistochemistry analysis, some studies suggest that ABBVs infection may cause nervous system disorders [5,7,10]. However, ABBVs have also been identified in birds without any apparent disease [8,12]. To evaluate the occurrence of ABBVs in wild avifauna in Poland, samples from dead wild birds (swans, geese and ducks) were tested for the presence of viral RNA. Approximately 35% of identified ABBVs from different regions of Poland was subjected to phylogenetic analysis to assess their genetic diversity and relationship with strains circulating in other countries.

## 2. Results

**Figure 1:**
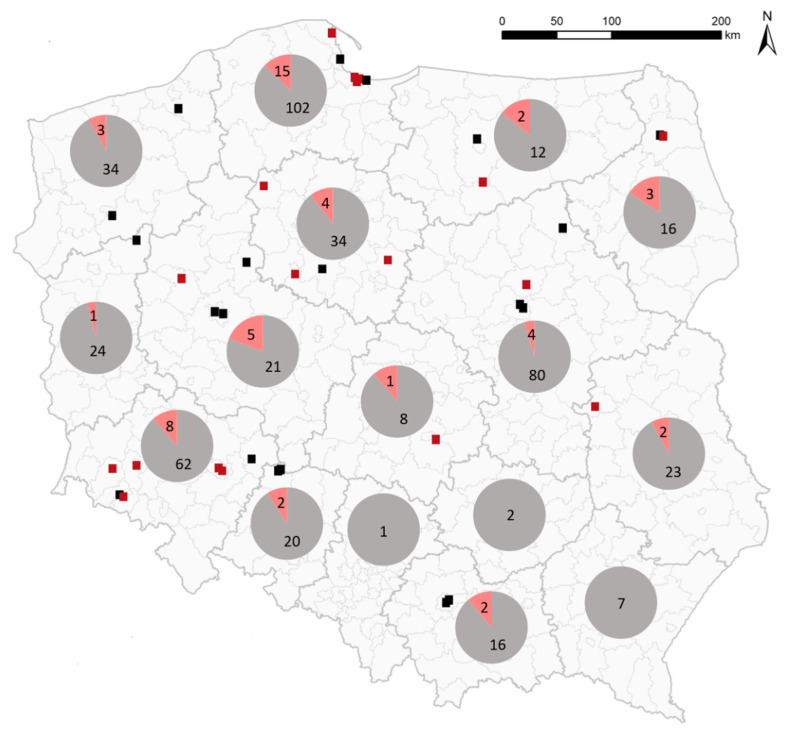
Location of ABBV-1 cases in Poland. Black and red points show locations of ABBV-1-positive birds. Red points indicate strains for which phylogenetic analysis was performed. Pie charts show the numbers and proportions of negative (grey) and positive (pink) birds in each province of Poland.

To investigate the presence of ABBVs in wild aquatic birds in Poland, samples from dead wild birds of the Anseriformes order were screened using a real time RT-PCR method targeting both ABBV-1 and ABBV-2. A total of 514 birds were tested, including 401 swans, 96 ducks and 17 geese. The presence of ABBV RNA was detected in 52 birds (10.1%), with positive samples originating only from swans (52/401; 13%) (Table 1) found in a total of 40 sites (Figure 1). Thirty-six ABBV-positive birds were identified as mute swans (*Cygnus olor*), one as whooper swan (*Cygnus cygnus*) and in 15 cases only the name of the genus was provided (*Cygnus* sp.) (Table 1).

To determine if the identified viruses were ABBV-1 or ABBV-2, fragments of viral genome were sequenced and compared to available sequences using BLAST search. Sequences were generated for samples from 48 birds and showed highest homology with ABBV-1. In four cases, probably due to low viral load (Ct values ~34), no sequences could be obtained.

Cases of ABBV-1 were detected in 13 out of 16 provinces of Poland (Figure 1). The differences between the number of infected birds and the number of locations resulted from finding multiple ABBV-positive birds at the same time and in the same place. Such cases were noted mainly in the Gdansk Bay in February–March 2021 where 14 ABBV-1-positive swans were found in seven locations during mass mortality events in that region caused by harsh weather conditions and highly pathogenic avian influenza (data unpublished).

Usually low Ct values (<25, occasionally between 11–15) were noted in organ pools, indicating high viral load, whereas low amounts of viral RNA were found in swabs (Ct > 30) (Table S1). Swabs constituted a small fraction of tested samples and only two birds were considered positive on the basis of results for combined oropharyngeal/cloacal swabs. Additionally, for five ABBV-positive birds, swabs were also available and tested positive in three cases (Table S1).

**Figure 2:**
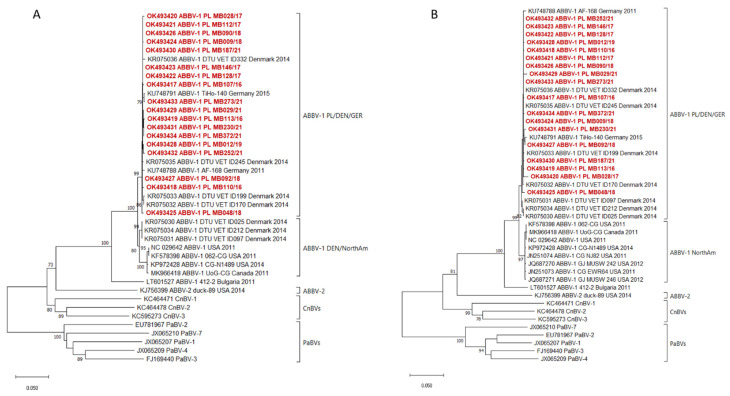
Phylogenetic trees for the nucleoprotein (**A**) and phosphoprotein (**B**) genes of ABBV-1 sequenced in this study (in red) and selected sequences from GenBank. Numbers next to the branches indicate bootstrap values (only values above 70% are shown). The scale bar represents the number of substitutions per site.

Eighteen ABBV-1-positive samples representing various spatiotemporal characteristics were selected for sequencing of longer genome fragments and phylogenetic analysis (Figure 1, Table S1). Conventional RT-PCRs employing four primer pairs (Table S2) were performed and followed by Sanger sequencing. Sequences of about 2300 bases covering N, X, P, M genes and a fragment of the G gene were generated. The identity of nucleotide sequences of Polish ABBV-1 strains ranged from 99.14% to 99.83%. Since most ABBV-1 sequences available in the GenBank database are incomplete, separate phylogenetic analyses for N and P genes with different sequence sets were performed with sequences of CnBVs and PaBVs used as outgroups. In both phylogenetic trees the Polish sequences formed a single cluster, also including sequences from Denmark and Germany (ABBV-1 PL/DEN/GER, Figure 2). The nucleotide identity within this cluster was 99.19–99.81% and 98.69–100% for the N and P gene, respectively. No specific grouping depending on the collection year or location could be distinguished. In the phylogenetic tree for the N gene, three ABBV-1 sequences from Denmark grouped separately and showed a higher relationship to sequences from North America than to those from Europe (ABBV-1 DEN/NorthAm, Figure 2A).

## 3. Discussion

The first reports suggesting that avian bornaviruses might be common in wild aquatic birds appeared in 2011 [5,8]. Since then, ABBVs have been identified in multiple species of the Anseriformes and Charadriiformes orders in North America and Europe [5,6,7,8,9,10,11,12,13]. In this study, we detected ABBV-1 in 52 swans, i.e., in 10% of all tested birds comprising swans, geese and ducks. The comparison of the detection rate with those observed in other studies is hindered due to varying inclusion criteria for tested birds and different sampling schemes (e.g., species, clinical status, location, sample and surveillance type). The fractions of positive anseriform birds reported in the studies from North America ranged from 78% in dead waterfowl with neurological disorders [7] to around 14–50% in apparently healthy birds that were hunted or euthanized [6,8,11]. Significantly lower detection rates were noted when testing swabs from live birds (2.9%) [8]. The percentage of ABBV-positive birds shown in the present study seems lower than ratios observed in North America but is higher than those in Denmark and Germany (2.1% and 0.9%, respectively) [12,13]. It should be stressed that the results obtained in the present study do not allow to calculate the prevalence of ABBV-1 in Poland as the data may be biased by non-representative, uneven sampling in different regions of Poland, targeted at dead individuals (passive surveillance). The detection rate reported might also be underestimated due to differences in organ sampling.

ABBV-1 was identified only in swans which constituted a vast majority of tested birds (78%). The lack of detection of ABBVs in ducks and geese might be a result of its lower prevalence in these groups and/or their underrepresentation in the total number of tested birds. However, similar results in terms of affected host species were obtained in Germany, where only mute swans showed positive results among all tested Anseriformes [13]. This finding may suggest that ABBV-1 may have a higher level of adaptation to swans. Nevertheless, additional epidemiological characteristics, such as the estimation of true prevalence in different waterfowl species (including possible seasonality) would shed more light on the epidemiology of ABBV-1 in wild avifauna and help to precisely identify the natural reservoir.

The geographical analysis showed a widespread distribution of ABBV-1 cases in the territory of Poland and although the paucity of surveillance data from other countries precludes the conclusion about the real distribution of the virus in Europe, the data obtained so far suggest that ABBV-1 is common in the European population of wild birds.

In the case of waterfowl, only the brain or other nervous system organs have been tested for the presence of ABBVs [5,6,7,8,9,10,11,12], thus the distribution of the virus in other tissues has not been investigated. Since the vast majority of the sample type available in the present study were mixtures of various organs, it was not possible to evaluate the tissue tropism. Bornaviruses are known of their strict neurotropism in incidental dead-end hosts, whereas in natural reservoir, the virus can be found in multiple tissue types but do not cause any clinical disorders [14]. In the present study, most tested samples included the brain, but positive results were obtained also for pools of other organs and for intestines tested separately in several cases, suggesting a pantropic nature of the infection. This is in congruence with investigations on the bornavirus distribution in tissues of infected parrots [15,16]. The level of oral and cloacal shedding in ABBV-infected birds has been shown to be low compared to the amounts of viral RNA found in brains, as the Ct values in swabs usually exceeded 30 [17,18]. Similar results were obtained for swabs analyzed in the present study.

All tested samples were collected from dead birds, but the sole detection of viral RNA is not sufficient enough to acknowledge the ABBV-1 infection as the cause of death. Due to the sample types available for this study, histopathological analysis could not be performed, nor was it possible to establish the clinical status of birds before their death. It is worth noting that almost half of the swans positive for ABBV-1 (46.2%) were also infected with highly pathogenic avian influenza virus (Table S1) which was the most probable cause of death. ABBV-1 has been detected in wild birds with neurologic or PDD-like diseases [7,10]. It was also suggested that ABBV-2 may cause vision disorders due to the presence of the virus in the retina [11]. Nevertheless, the virus has also been detected in birds without any overt clinical manifestation [8,12,13,17]. Therefore, further studies on the pathobiological features, such as pathogenicity (including the ability to cause death), virus distribution in organs or the extent of tissue damage, are needed to elucidate the significance of the ABBV-1 infection in waterfowl and help to identify asymptomatic carriers and birds that succumb to infection and could serve as sentinels.

Phylogenetic analysis of 18 Polish strains showed their close relationship with ABBV-1 identified in Denmark and Germany and their distinctiveness from viruses from North America, which is in agreement with a previous study [13]. A relatively low genetic diversity was observed for the identified ABBV-1 strains as the nucleotide identity of the sequenced genome fragments was above 99%. Comparison of sequences from Poland, Denmark and Germany, forming one cluster of strains detected within the last 10 years, revealed a similar level of nucleotide identity which demonstrates high conservativeness of the ABBV-1 genome. Although the existence of separate geographical lineages of ABBV-1 is explicable by the separation of the Eurasian and North American population of wild waterfowl and is well known for other viruses (e.g., avian influenza viruses). However, it should be noted that several ABBV-1 strains from Denmark clustered with North American ABBV-1 in the N gene tree suggests a possible intercontinental virus exchange. Scarcities of sequences in the database disable more accurate insight into the genetic diversity of currently circulating strains both on a global and local level. Sequencing of newly identified ABBVs would allow a more comprehensive analysis of the evolutionary characteristics of this virus in the wild bird reservoir.

## 4. Materials and Methods

### 4.1. Samples from Wild Birds

Samples used in the study originated from dead wild birds and were collected in the frame of passive surveillance for avian influenza in 2016–2021. The majority of samples were collected between late autumn and early spring. A total of 514 birds of the Anseriformes order were tested, including swans (*n* = 401), ducks (*n* = 96) and geese (*n* = 17). Species of tested birds are listed in Table 1. The main sample type available were pools of organs from individual birds, and in some cases oropharyngeal and/or cloacal swab and the brain were tested. The most frequently collected organs included intestines, liver, spleen, lungs and brain. Organ pools were prepared as 20% homogenates in phosphate buffered saline (PBS). Swabs were immersed in PBS (1 mL/swab).

### 4.2. Detection of ABBVs RNA with Real Time RT-PCR

The RNA was extracted manually using RNeasy Mini Kit (Qiagen, Hilden, Germany) or in an IndiMag 48 s robot using IndiMag Pathogen Kit (Indical Biosciences, Leipzig, Germany). A real time RT-PCR assay was developed with primers and probe targeting a conserved region of the genome coding for the X and P proteins (ABBV-F: 5′-GAAGACTSAATGGCATCTCGAC-3′; ABBV-R: 5′-GGTTGTGTCAGRGCATCTGG-3′; ABBV-pro: 5′-FAM-ACCCGCAGACAGCACGTCGCA-BHQ1-3′). The oligonucleotides were designed to detect both ABBV-1 and ABBV-2 based on sequences available in the GenBank database. The tests were performed using QuantiTect Probe RT-PCR Kit (Qiagen, Hilden, Germany) and each reaction contained 1× QuantiTect Probe RT-PCR Master Mix, 0.8 µM of each primer, 0.4 µM probe, 1× QuantiTect RT Mix and 5 µL of RNA in a total volume of 25 µL. An ABI 7500 real time PCR machine was used with the following thermal profile: 50 °C for 30 min, 95 °C for 15 min, 40 cycles of 94 °C for 15 s and 58 °C for 30 s. Samples with Ct value of < 35 were considered positive.

### 4.3. Sequencing and Phylogenetic Analysis

Eighteen samples positive in real time RT-PCR were selected for sequencing of a genome fragment of about 2300 bases and phylogenetic analysis. The selected subset of samples represented various locations and time of collection. Sequences were obtained using the Sanger method following amplification of overlapping fragments in conventional RT-PCR. Four primer pairs were designed and used (Table S2), yielding PCR products covering a fragment of about 2300 bases comprising complete sequences of N, X, P, M genes and partial sequence of the G gene. Reactions were performed using OneStep RT-PCR Kit (Qiagen, Hilden, Germany) with 0.8 µM primers, 1× OneStep RT-PCR Buffer, 1 µL of Enzyme Mix, 1 µL of dNTP mix and 2.5 µL of RNA in a total volume of 25 µL. The cycling conditions were as follows: 50 °C for 30 min, 95 °C for 15 min, 40 cycles of 95 °C for 30 s, 54 °C for 30 s, 72 °C for 1 min and a final stage of 72 °C for 5 min. The reaction products were separated in 1.5% agarose gel and verified for the presence of the expected band size. Sanger sequencing was performed in both directions using BigDye Terminator v3.1 Cycle Sequencing Kit (ThermoFisher Scientific, Waltham, MA, USA) in 3500 Genetic Analyzer (Applied Biosystems, Foster City, CA, USA). Chromatograms were analyzed and assembled in SeqScape Software v2.7 (Applied Biosystems). Phylogenetic analysis was performed in MEGA X [19] using the Neighbor-Joining method and Jukes–Cantor model with 1000 bootstrap replication. Sequences of other avian bornaviruses available in the GenBank database were included in the analysis. The ~2300-base fragments of eighteen Polish ABBV-1 strains used in the phylogenetic analysis were deposited in GenBank with accession nos. OK493417-OK493434. Additionally, to determine if the remaining 34 ABBVs detected in the present study were ABBV-1 or ABBV-2, sequencing of the real time RT-PCR amplicons (~130 bp) or the conventional RT-PCR product obtained with primers ABBV-1F/ABBV-1R (~800 bp) (Table S2) was undertaken. Sequences were generated for 30 viruses and compared to available sequences using BLAST search.

## Figures and Tables

**Table 1:** A summary of species and number of birds tested for the presence of ABBVs RNA in Poland.

Species	Number of Tested Birds	Number of ABBV-Positive Birds
mute swan (*Cygnus olor*)	220	36
whooper swan (*Cygnus cygnus*)	2	1
black swan (*Cygnus atratus*)	9	0
swan (*Cygnus* sp.) ^1^	170	15
mallard (*Anas platyrhynchos*)	48	0
long-tailed duck (*Clangula hyemalis*)	11	0
garganey (*Spatula querquedula*)	3	0
wild duck ^2^	31	0
other ducks ^3^	3	0
bean goose (*Anser fabalis*)	7	0
tundra bean goose (*Anser serrirostris*)	3	0
greylag goose (*Anser anser*)	3	0
white-fronted goose (*Anser albifrons*)	1	0
wild goose ^2^	3	0
Total	514	52

^1^ Only genus name provided during sample submission; ^2^ Species unspecified; ^3^ Includes a tufted duck, Mandarin duck and ferruginous duck.

## Data Availability

The data presented in this study are available in the main text or in the supplementary materials. Sequences are available in GenBank (https://www.ncbi.nlm.nih.gov/genbank/).

## Supplementary Materials

The following are available online at https://www.mdpi.com/article/10.3390/pathogens11010098/s1, Table S1. List of ABBV-positive birds; Table S2. Primers used for amplification of fragments of ABBV-1 genome.
